# Radiomics Approach for Prediction of Recurrence in Non-Functioning Pituitary Macroadenomas

**DOI:** 10.3389/fonc.2020.590083

**Published:** 2020-12-18

**Authors:** Yang Zhang, Ching-Chung Ko, Jeon-Hor Chen, Kai-Ting Chang, Tai-Yuan Chen, Sher-Wei Lim, Yu-Kun Tsui, Min-Ying Su

**Affiliations:** ^1^ Department of Radiological Sciences, University of California, Irvine, Irvine, CA, United States; ^2^ Department of Medical Imaging, Chi-Mei Medical Center, Tainan, Taiwan; ^3^ Department of Health and Nutrition, Chia Nan University of Pharmacy and Science, Tainan, Taiwan; ^4^ Department of Radiology, E-DA Hospital, I-Shou University, Kaohsiung, Taiwan; ^5^ Graduate Institute of Medical Sciences, Chang Jung Christian University, Tainan, Taiwan; ^6^ Department of Neurosurgery, Chi-Mei Medical Center, Chiali, Tainan, Taiwan; ^7^ Department of Nursing, Min-Hwei College of Health Care Management, Tainan, Taiwan

**Keywords:** radiomics, MRI, pituitary, macroadenoma, recurrence

## Abstract

**Objectives:**

A subset of non-functioning pituitary macroadenomas (NFPAs) may exhibit early progression/recurrence (P/R) after surgical resection. The purpose of this study was to apply radiomics in predicting P/R in NFPAs.

**Methods:**

Only patients who had undergone preoperative MRI and postoperative MRI follow-ups for more than 1 year were included in this study. From September 2010 to December 2017, 50 eligible patients diagnosed with pathologically confirmed NFPAs were identified. Preoperative coronal T2WI and contrast-enhanced (CE) T1WI imaging were analyzed by computer algorithms. For each imaging sequence, 32 first-order features and 75 texture features were extracted. Support vector machine (SVM) classifier was utilized to evaluate the importance of extracted parameters, and the most significant three parameters were used to build the prediction model. The SVM score was calculated based on the three selected features.

**Results:**

Twenty-eight patients exhibited P/R (28/50, 56%) after surgery. The median follow-up time was 38 months, and the median time to P/R was 20 months. Visual disturbance, hypopituitarism, extrasellar extension, compression of the third ventricle, large tumor height and volume, failed optic chiasmatic decompression, and high SVM score were more frequently encountered in the P/R group (*p* < 0.05). In multivariate Cox hazards analysis, symptoms of sex hormones, hypopituitarism, and SVM score were high risk factors for P/R (*p* < 0.05) with hazard ratios of 10.71, 2.68, and 6.88. The three selected radiomics features were T1 surface-to-volume radio, T1 GLCM-informational measure of correlation, and T2 NGTDM-coarseness. The radiomics predictive model shows 25 true positive, 16 true negative, 6 false positive, and 3 false negative cases, with an accuracy of 82% and AUC of 0.78 in differentiating P/R from non-P/R NFPAs. For SVM score, optimal cut-off value of 0.537 and AUC of 0.87 were obtained for differentiation of P/R. Higher SVM scores were associated with shorter progression-free survival (*p* < 0.001).

**Conclusions:**

Our preliminary results showed that objective and quantitative MR radiomic features can be extracted from NFPAs. Pending more studies and evidence to support the findings, radiomics analysis of preoperative MRI may have the potential to offer valuable information in treatment planning for NFPAs.

## Introduction

Pituitary adenomas constitute 10–15% of all intracranial tumors (1), the majority being non-functioning pituitary adenomas (2, 3). The most common presentation is the macroadenoma, which is defined as a tumor larger than 10 mm in size. Non-functioning pituitary macroadenomas (NFPAs) may cause bitemporal hemianopia resulting from optic chiasm compression due to mass effect. Hypopituitarism is observed in some patients due to tumor compression of normal pituitary glandular tissue. According to 2017 WHO classification system, pituitary tumors are formally classified as adenoma, carcinoma, or blastoma (4). Although more than 90% of NFPAs are diagnosed as benign tumors, 25–55% of these tumors may undergo early progression/recurrence (P/R) after surgical resection (5–8). Gross-total resection (GTR) *via* a transsphenoidal approach (TSA) is the optimal method of treatment for NFPAs in current clinical practice. However, complete resection is often difficult to achieve for large solid tumor with extrasellar extension (9). Although adjuvant radiotherapy (RT) is implemented in some institutions in attempts to minimize postoperative P/R in NFPAs, this approach may result in progressive pituitary insufficiency and other long-term complications (10).

Conventional MR imaging findings such as cavernous sinus invasion, tumor size, and absence of tumor apoplexy have been reported as important parameters related to P/R in NFPAs. However, the abovementioned parameters are subjective to significant inter-observer variation (11, 12). Radiomics analysis is recently emerging as a comprehensive quantitative method for the evaluation of various clinical diseases (13–15). The extracted imaging features have been shown to reveal visually imperceptible information extending beyond radiology to histopathology. Several studies even suggest that radiomics may be able to provide valuable predictors regarding diagnosis, prognosis, and thus aid in therapeutic planning in brain tumors (13, 16–18).

In regard to the application in NFPAs, radiomics has been used in the evaluation of tumor subtypes, consistency, ki-67 proliferation indices, and cavernous sinus invasion (18–22), but rarely for the prediction of clinical outcomes (23). The purpose of this study was to investigate the role of radiomics features extracted from segmented tumor sampling for the prediction of P/R in NFPAs.

## Materials and Methods

### Ethics Statement

This study was approved by our Institutional Review Board (IRB no. 10902-009). Written consent was waived because the retrospective nature of this project does not influence the health-care of the included patients. All patients’ medical records and imaging documentations were anonymized and de-identified prior to analysis.

### Patient Selection

The inclusion criteria of this study were patients diagnosed with benign NFPAs by pathological confirmation, complete and good imaging quality of preoperative brain MRIs, and postoperative follow-up MRIs more than 1 year after treatment. Patients with clinical, biochemical, and histopathological evidence of hormone hypersecretion were excluded. According to studies by Brochier et al. (11) and Hong et al. (24), diagnosis of prolactinoma is considered unlikely if the prolactin levels were below 100 mg/L, a conclusion thereafter confirmed by immunocytochemical studies. Patients who received postoperative adjuvant RT before P/R were also excluded. From September 2010 to December 2017, 50 patients (29 men, 21 women, age 19–80 years; median age, 52 years) were identified for this study in accordance with the abovementioned inclusion and exclusion criteria. Forty-eight patients underwent surgery performed by TSA, and craniotomy was performed in two patients due to large tumor sizes. The median follow-up duration for all patients was 38 months (range from 12 to 115 months). In 28 patients with P/R, the median time to P/R was 20 months (range from 6 to 67 months). Clinical and biochemical data were also obtained from medical records.

### Extent of Resection and Progression/Recurrence

The extent of surgical resection was determined by review of postoperative MRI by a neuroradiologist (C-CK) and a neurosurgeon (S-WL). According to published literature (25), GTR was defined as lesion resection with a residual tumor volume of less than 10% of its original size. In contrast, subtotal tumor resection (STR) was defined as the presence of residual lesion more than 10% of its original volume. For determining P/R in NFPAs, pretreatment and postoperative MR images were evaluated by two experienced neuroradiologists (C-CK with 6 years of experience and T-YC with 18 years of experience), both of whom were blinded to the clinical and imaging outcomes of the studied population. P/R was defined as tumor recurrence after GTR or enlargement of residual tumor after STR observed on postoperative contrast-enhanced (CE) T1WI. The threshold of P/R was defined as a more than 2-mm increase in size of residual tumor in at least one dimension when compared with postoperative MRI studies (11, 26). Inter-observer reliability in the determination of P/R was obtained *via* a Cohen k value of 0.9. In equivocal cases, judgment was made *via* consensus. On preoperative MR images, cavernous sinus invasion (Knosp classification) (27) and extrasellar extension (Hardy’s classification) (28) were determined on coronal T2WI and CE T1WI.

### Imaging Acquisition

Preoperative brain MRI images were acquired with a 1.5-T (Siemens, MAGNETOM Avanto) (n = 19), 1.5-T (GE Healthcare, Signa HDxt) (n = 17), or a 3-T (GE Healthcare, Discovery MR750) (n = 14) MR scanner equipped with eight-channel head coils in each machine. Scanning protocols include axial and sagittal spin echo T1-weighted imaging (T1WI), axial and coronal fast spin echo T2- weighted imaging (T2WI), axial fluid attenuated inversion recovery (FLAIR), and axial T2*- weighted gradient-recalled echo (GRE). Dynamic contrast-enhanced (CE) coronal T1WI images with a small field of view through the pituitary gland as well as coronal and sagittal CE T1WI with fat saturation were performed after intravenous administration of 0.1 mmol/kg of body weight of gadobutrol or gadoterate meglumine. Detailed imaging parameters in the MR scanners were described in **Supplementary File 1**.

### Tumor Segmentation

**Figure 1:**
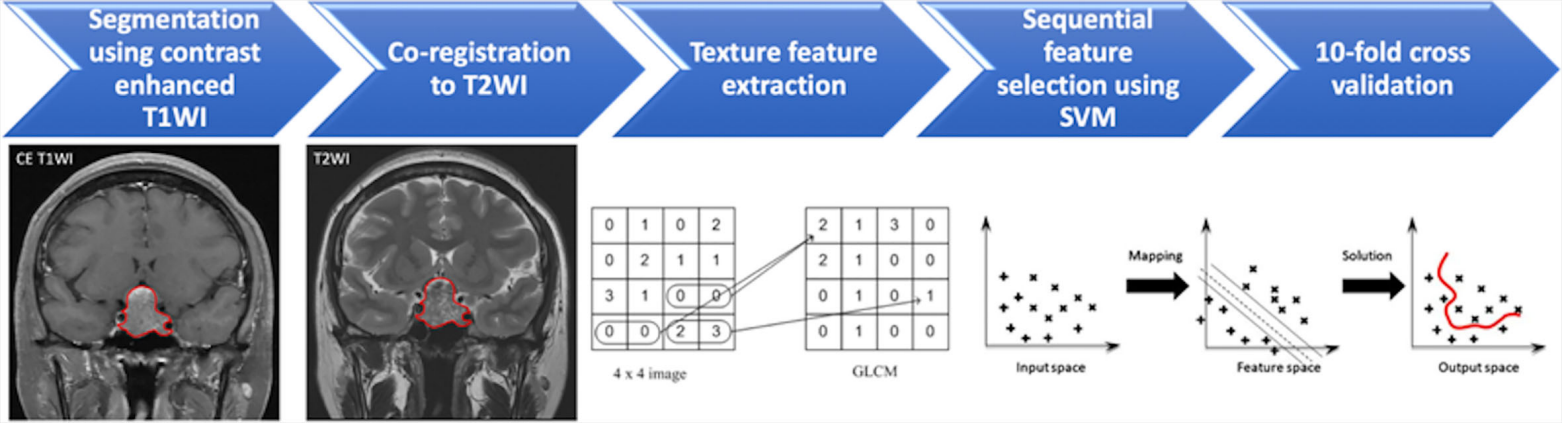
Flowchart of the analytical process for prediction of progression/recurrence (P/R) in non-functioning pituitary macroadenomas (NFPAs). The NFPA (red outline) is segmented on coronal contrast-enhanced (CE) T1WI and then mapped to the coronal T2WI. On each set of images, a total of 107 imaging features, including 32 first order features and 75 textural features, are extracted. The most important three features are selected by sequential feature selection and support vector machine (SVM) classifiers to build the prediction model. A 10-fold cross validation method is applied to test the model performance.

Because both T2WI and CE T1WI are associated with cavernous sinus invasion, histopathologic subtypes, tumor consistency, and therapeutic response in pituitary tumors (18, 19, 21, 29–31), they were analyzed in our study. **Figure 1** shows the flowchart in the process of analysis. Tumor segmentation was performed on coronal CE T1WI with MATLAB 2018b software (32). In image pre-processing, the slices were resampled to isotropic 3D rendering. Then the pixel intensities inside the 3D rendered ROIs were normalized to mean of 0 and standard deviation of 1. For each lesion, the operator places an initial rectangular region of interest (ROI) on the image to locate the tumor as well as select the beginning and ending slices containing the lesion. Subsequently, the fuzzy c-mean (FCM) clustering algorithm was applied to segment the lesion ROI on each image slice (33). In cases of under- or over-segmentation, manual correction was performed. After segmentation/correction was performed, the ROIs from all imaging slices containing the particular tumor were combined. The 3D connected-component labeling was then applied to remove scattered voxels not connected to the main lesion. The hole-filling algorithm was applied to include all voxels contained within the main ROI labeled as non-lesion. The segmented tumor mask was transferred onto corresponding coronal T2WI by using affine transformation with linear interpolation. This process was conducted by FMRIB’s Linear Image Registration Tool (FLIRT) (34).

### Texture Feature Extraction and Selection

Within segmented tumor on CE T1WI and T2WI, 107 imaging features, including 32 first order features and 75 textural features were extracted on each modality by Python 3.75 software (35) (**Figure 1**). Filters were not used in the feature extraction process. Because some small NFPAs may be inseparable from surrounding normal pituitary tissue, boundary pixels of tumor masks on each slice were removed by binary erosion to ensure only tumorous tissues were included in the ROI (32). Lengths of 0.25 and 0.5 cm were used to determine the outer shells of the boundary pixels to be removed. Therefore, three tumor ROIs were obtained: original masks, original mask with 0.25 cm erosion, and original mask with 0.5 cm erosion. For each of the abovementioned tumor ROIs, a total of 214 features were extracted from CE T1WI and T2WI.

To evaluate the importance of these features in the differentiation between patients with and without P/R, the sequential feature selection process was implemented *via* constructing multiple support vector machine (SVM) classifiers by MATLAB 2018b software (32). In this process, SVM with Gaussian kernel was used as the objective function to test the performance of models built with a subset of features (36, 37). In the beginning, an empty candidate set was presented, and features were sequentially added. The 10-fold cross validation method was applied to test the model performance (38). For each iteration, the training process was repeated 1,000 times to explore the robustness of each feature. At the conclusion of each iteration, the feature which results in the best performance was added into the candidate set. In this instance, we use 10^−6^ as the termination tolerance for the objective function value. Once the addition of features no longer meets the criterion, cessation of the selection process ensues.

Besides, the SVM score was calculated for each patient based on the selected features as described below.

$$f\left(x\right)=\;\sum_{n=1}^{N}w_{n}y_{n}G(x_{n},x)+b$$

where x was the input features, N was the length of support vector. *y_n_* and *x_n_* were the entries of the supporting vector. Wn was the parameter and b was the bias. G(*x_n_*, *x*) was the Gaussian kernel function which indicated the dot product in the predictor space between x and the support vectors (33). Here,

$$G(x_{n},\;x)=e^{-{∥x_{n}-x∥}^{2}}$$

### Statistical Analysis

Statistical analyses were performed using SPSS for Windows (V.24.0, IBM, Chicago, IL, USA). For the evaluation of clinical parameters and conventional MR imaging, Chi-square (or Fisher exact test) and Mann-Whitney U tests were performed for categorical and continuous data, respectively. The true positive (TP), true negative (TN), false positive (FP), false negative (FN), accuracy, and area under the receiver operating characteristic curve (ROC) curve (AUC) in prediction models of different tumor masks were calculated. ROC analysis of SVM scores was performed to obtain the optimal cut-off value. Further, Kaplan-Meier analysis based on cut-off value of SVM score was used to evaluate the progression-free survival (PFS), and log-rank test was used to assess the significance. Cox proportional hazard model with univariate and multivariate analysis was performed to determine independent predictors of P/R. Variables with a *p* < 0.05 in univariate analysis were brought forward to the multivariate analysis. For multivariate analyses and all other statistical analyses, *p* < 0.05 were considered statistically significant.

## Results

### Clinical Data and Conventional MRI Findings

**Figure 2:**
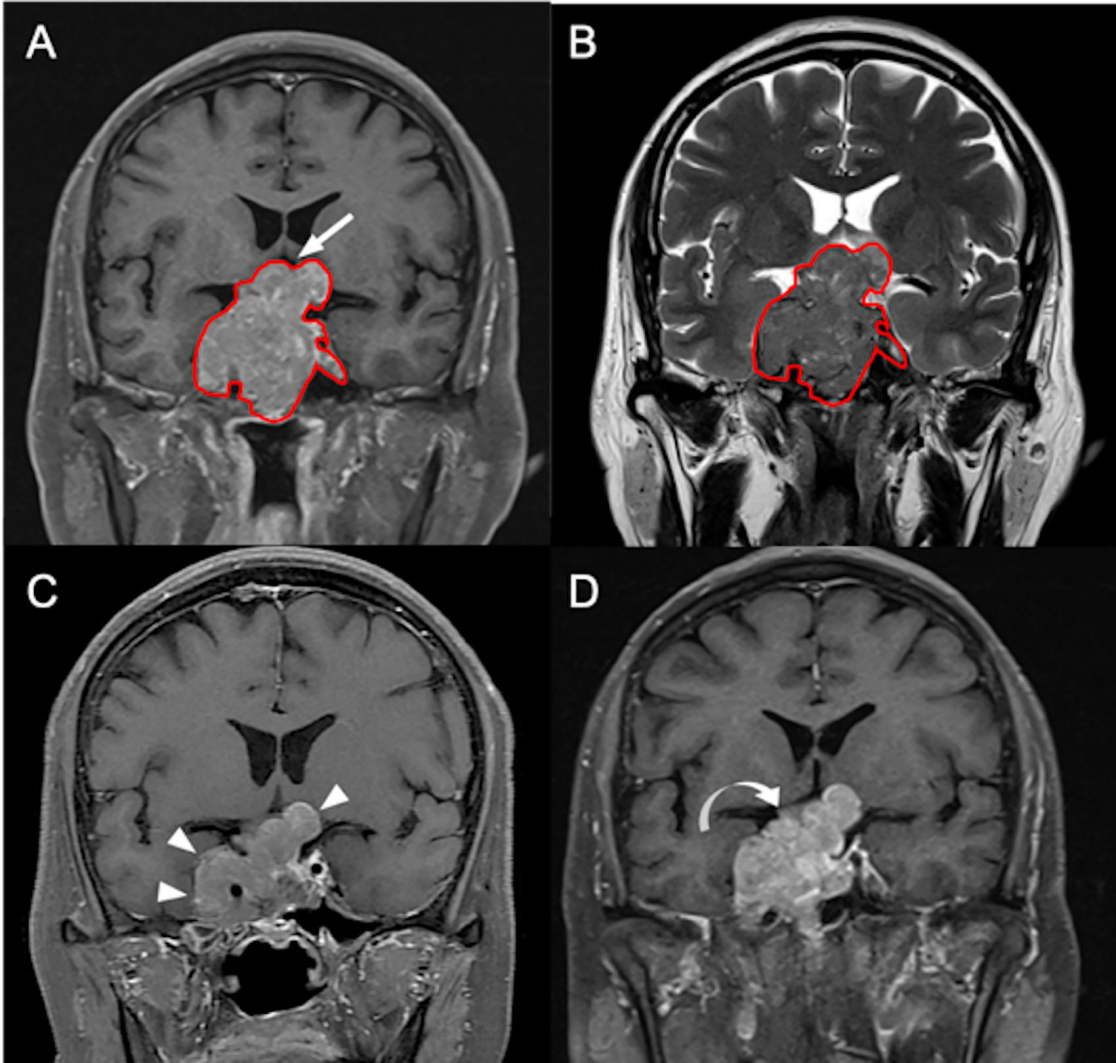
A 55-year-old male patient with left hemianopia and pathologically confirmed NFPA. **(A)** Coronal CE T1WI shows an enhancing sellar tumor (red outline) with upward suprasellar extension and bilateral cavernous sinus invasion, causing compression of the optic chiasm and the third ventricle (arrow indicates area of optic chiasm and third ventricle). **(B)** The tumor (red outline) is segmented on coronal CE T1WI **(A)** and then mapped to the coronal T2WI **(B)**. **(C)** Improvement of blurred vision after subtotal tumor resection *via* transsphenoidal approach is clinically documented, and the maximum height of the residual tumor (arrowheads) measured from coronal CE T1WI is 38 mm. **(D)** Recurrent visual deterioration with enlargement of the residual tumor (curved arrow) with maximum height up to 48 mm is observed 19 months after surgical resection.

The clinical data and conventional MRI findings were summarized in **Table 1**. P/R was diagnosed in twenty-eight (28/50, 56%) patients. No statistical difference was found between the extent of tumor resection and P/R (*p* = 0.157). Visual disturbance, hypopituitarism, extrasellar extension, compression of the 3rd ventricle, large tumor height and volume, and high SVM score were more frequently observed in the P/R group (*p* < 0.05) (**Figure 2**). In multivariate Cox proportional hazards analysis (**Table 2**), symptoms of sex hormones, hypopituitarism, and SVM score were high risk factors for P/R (*p* < 0.05) with hazard ratios of 10.71, 2.68, and 6.88.

**Table 1:** The clinical data and conventional MR imaging of non-functioning pituitary macroadenomas (NFPAs) with and without progression/recurrence (P/R).

	P/R	Non-P/R	p
**Number of patients**	28	22	
**Sex**			0.111
Male	19 (67.9%)	10 (45.5%)	
Female	9 (32.1%)	12 (54.5%)	
**Age (y)**	53.5 (44, 63)	42 (23.5, 60.5)	0.089
**Clinical symptoms**			
Visual disturbance	26 (92.9%)	13 (59.1%)	0.006*
Headache	8 (28.6%)	11 (50%)	0.121
Symptoms of sex hormones (decreased libido, sexual dysfunction, and/or amenorrhea/oligomenorrhea)	5 (17.9%)	1 (4.5%)	0.211
Incidental	2 (7.1%)	4 (18.2%)	0.385
**Hypopituitarism**			0.047*
No	12 (42.9%)	17 (77.3%)	
Single	8 (28.6%)	3 (13.6%)	
Multiple	8 (28.6%)	2 (9.1%)	
**Hyperprolactinemia**	10 (35.7%)	6 (27.3%)	0.525
**Extent of surgical resection**			0.157
Gross-total resection (GTR)	3 (10.7%)	6 (27.3%)	
Gross-total resection (STR)	25 (89.3%)	16 (72.7%)	
**Successful chiasmatic decompression**	9 (32.1%)	17 (77.3%)	0.002*
**Cavernous sinus invasion** **(Knosp classification)**			0.077
Grade 1–2	18 (64.3%)	19 (86.4%)	
Grade 3–4	10 (35.7%)	3 (13.6%)	
**Extrasellar extension** **(Hardy’s classification)**			0.045*
Grade 1–2	17 (60.7%)	19 (86.4%)	
Grade 3–4	11 (39.3%)	3 (13.6%)	
**Compression of optic chiasm**	27 (96.4%)	17 (77.3%)	0.075
**Compression of the third ventricle**	21 (75%)	9 (40.9%)	0.015*
**Hydrocephalus**	2 (7.1%)	1 (4.5%)	1
**Giant (>40 mm)**	9 (32.1%)	2 (9.1%)	0.085
**Maximum tumor height (mm)**	35.5 (27.5, 43.5)	18 (10, 26)	<0.001*
**Tumor volume (cm^3^)**	12.3 (4.4, 20.1)	2.7 (1.2, 8)	<0.001*
**SVM score**	0.999 (0.960, 1.040)	0.030 (−0.241, 0.301)	<0.001*

Continuous variables were presented as median and interquartile range (IQR).

*Statistical difference (p < 0.05).

**Table 2:** Cox proportional hazards analysis for P/R.

	Univariate Analysis		Multivariate Analysis	
	HR (95% CI) for P/R	*p*	HR (95% CI) for P/R	*p*
Sex (fraction male)	1.980 (0.861, 4.551)	0.108		
Age (years)	1.020 (0.996, 1.045)	0.098		
Visual disturbance	3.378 (0.797, 14.311)	0.098		
Headache	0.825 (0.361, 1.889)	0.649		
Symptoms of sex hormones	5.792 (2.000, 16.777)	0.001*	10.713 (2.884, 39.800)	< 0.001*
Incidental	0.642 (0.152, 2.721)	0.548		
Hypopituitarism	2.772 (1.27, 6.052)	0.01*	2.680 (1.121, 6.49)	0.027*
Hyperprolactinemia	1.162 (0.504, 2.679)	0.724		
Non-GTR	1.311 (0.389, 4.418)	0.662		
Successful chiasmatic decompression	0.400 (0.180, 0.888)	0.024*	1.012 (0.404, 2.537)	0.979
Cavernous sinus invasion (Knosp grades 3–4)	1.460 (0.647, 3.295)	0.363		
Extrasellar extension (Hardy’s grade 3–4)	1.728 (0.792, 3.768)	0.169		
Compression of optic chiasm	3.354 (0.454, 24.766)	0.236		
Compression of the third ventricle	1.769 (0.74, 4.228)	0.199		
Hydrocephalus	2.117 (0.483, 9.275)	0.32		
Giant NFPA (>40 mm)	2.964 (1.277, 6.883)	0.011*	2.061 (0.562, 7.560)	0.276
Maximum tumor height (mm)	1.164 (1.046, 1.296)	0.005*	1.060 (0.889, 1.264)	0.518
Tumor volume (cm^3^)	1.031 (1.011, 1.051)	0.002*	0.988 (0.954, 1.024)	0.506
SVM score	10.037 (2.252, 44.740)	0.002*	6.879 (1.328, 35.621)	0.022*

*Statistical difference (p < 0.05).

### Radiomics Approach for Prediction of P/R

**Figure 3:**
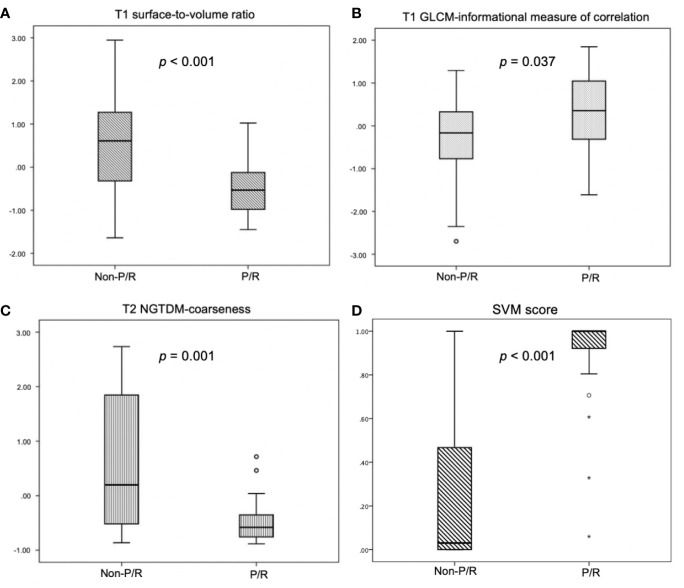
Box plot of **(A)** T1 surface-to-volume ratio, **(B)** T1 GLCM-informational measure of correlation, **(C)** T2 NGTDM-coarseness, and **(D)** SVM score for prediction of P/R in NFPAs. Statistically significant differences (*p* < 0.05) (Mann-Whitney U test) in the selected features and SVM score are observed. Boxes indicate the interquartile range (IQR), and whiskers indicate the range. The horizontal line represents the median in each box. Circles represent outliers, which are defined as distances greater than 1.5 times the IQR below the first quartile or above the third quartile. Stars represent extreme values, defined as distances greater than three times the IQR below the first quartile.

In radiomics analyses, the most important three parameters selected by the final SVM model for the prediction of P/R were: T1 surface-to-volume radio, T1 GLCM-informational measure of correlation, and T2 NGTDM-coarseness, and all show significant differences (Mann-Whitney U test) (**Figure 3**). The reproducibility of ROI-based radiomics feature was good between two readers, and the intra-class correlation coefficients (ICCs) of the three imaging features were 0.90, 0.80, and 0.87 respectively.

**Figure 4:**
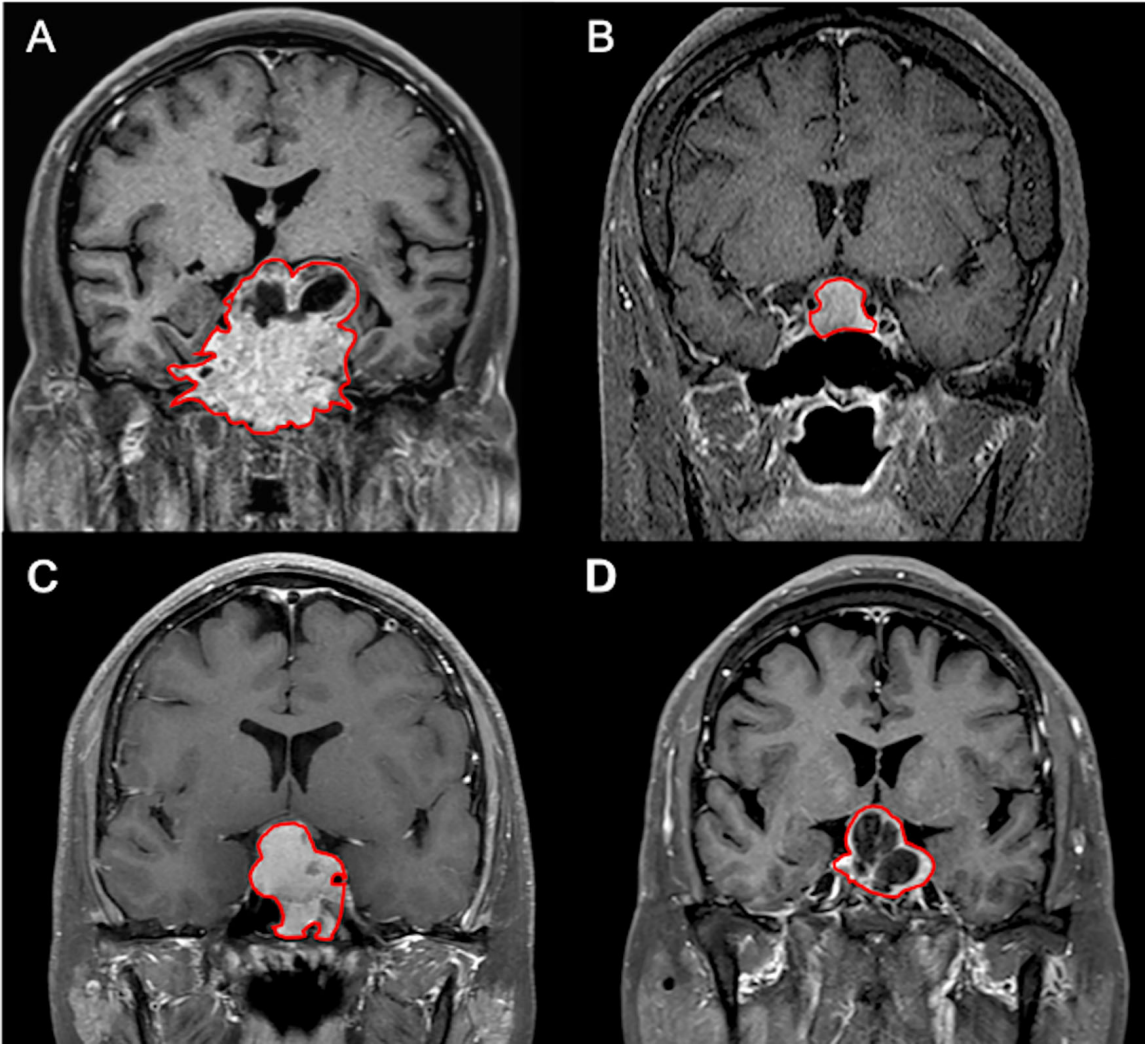
Examples of NFPAs (red outline) on coronal CE T1WI showing **(A)** true positive (TP), **(B)** true negative (TN), **(C)** false positive (FP), and **(D)** false negative (FN) results in the prediction model. **(A)** In the TP group, larger tumor sizes with more surrounding bone invasion are observed. **(B)** In contrast, smaller tumor sizes without bone invasion are found in most TN cases. **(C)** Most FP cases showed relatively homogeneous contrast enhancement without apoplexy or cystic change. **(D)** Two of the three FN cases exhibit macrocystic components or apoplexy.

**Figure 5:**
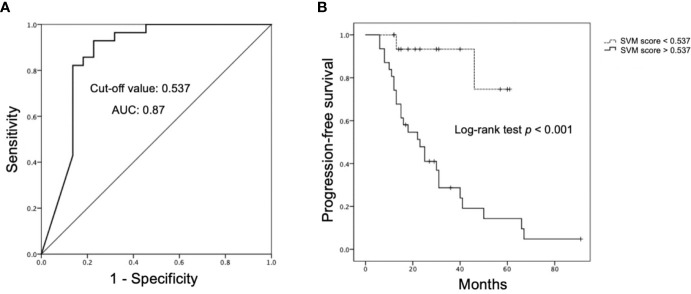
Receiver operating characteristic (ROC) and Kaplan-Meier survival curves of SVM score. **(A)** ROC curve of SVM score for prediction of P/R in NFPAs, with optimal cut-off value of 0.537 and AUC of 0.87. **(B)** Kaplan-Meier survival curves showing significant difference (*p* < 0.05) (Log-tank test) in overall trend of progression-free survival based on cut-off value of SVM score.

The SVM classification results by the original mask shows 25 TP, 16 TN, 6 FP, and 3 FN cases (**Figure 4**) with accuracy of 82% and AUC of 0.78 (**Table 3**). The optimal cut-off value of SVM score for differentiation of P/R was 0.537, with AUC of 0.87 (**Figure 5**). When tumor progression trends were compared, patients with high SVM score (more than the cut-off value of 0.537) were found to exhibit shorter PSF (*p* < 0.001) (**Figure 5**).

**Table 3:** Performance in prediction models with and without binary erosions.

	TP	TN	FP	FN	Accuracy	AUC
**Original mask**	25	16	6	3	82%	0.78
**With 0.25* cm* erosion**	24	16	6	4	80%	0.80
**With 0.5* cm* erosion**	24	17	5	4	82%	0.79

## Discussion

In this study, we developed a radiomics model to predict P/R in NFPAs. Three tumor ROIs, including the original mask and mask with binary erosions, were used. Three features were selected by SVM algorithm to build the final predication model: two from CE T1WI and one from T2WI. The overall accuracy was 82% with AUC of 0.78, and there was no significant difference amongst the three tumor ROIs methods. This study also calculated SVM score for prediction of P/R in NFPAs, and patients with higher SVM score were found to exhibit shorter PSF. In multivariate Cox hazards analysis, symptoms of sex hormones, hypopituitarism, and SVM score were high risk factors of P/R in NFPAs.

Although more than 90% of NFPAs are benign according to the 2017 WHO classification system (4), 25–55% may exhibit early P/R within 5 years after surgical resection (5–8). The Ki-67 index, mitotic count, and tumor invasion are all associated with aggressive clinical behavior in NFPAs (4). However, the invasive growth of NFPAs is not clearly defined in the WHO criteria, and it is usually underestimated if the corresponding information from MR imaging is not taken into consideration (8). A meta-analysis including 143 studies by Roelfsema et al. (8) showed that postoperative hormone concentration is an important predictor for P/R in functioning pituitary adenomas, but no specific factor is found for NFPAs.

Recently, low apparent diffusion coefficient (ADC) on diffusion-weight MR imaging (DWI), indicating a higher cellular density, is reported to be associated with tumor progression in NFPAs (26, 39). However, the ADC values may be affected by susceptibility artifacts from blood products because of apoplexy or necrosis; therefore, they could only be measured for solid NFPAs without hemorrhage or cystic changes (9, 26, 40). The radiomics analysis can be applied to the whole tumor to obtain reproducible, objective, and quantitative data from different imaging sequences, thus providing a more comprehensive method in the approach of various acquired information (13–15). For application of radiomics in pituitary tumors, Saha et al. (41) reported a review article including 16 studies from the past 10 years (2009–2019). Ten of these studies were undertaken from 2018 to 2019, most of which utilized single-centered, retrospective data, semi-automatic pipelines, and binary classifications as in our study. Zhang et al. (19) applied preoperative radiomics to distinguish null cell adenomas from other subtypes in NFPAs with AUC of 0.8 to 0.83. Rui et al. (18), Zeynalova et al. (31), and Cuocolo et al. (42) used preoperative radiomics texture and histogram analysis to predict consistency in pituitary macroadenomas with AUCs of 0.836, 0.71, and 0.99 respectively. Fan et al. (20, 29) and Kocak et al. (30 **)** used radiomics to predict response to radiotherapy and somatostatin analogues in acromegaly with AUCs of 0.96 and 0.845 respectively. Niu et al. (21) used radiomics to predict cavernous sinus invasion in NFPAs with AUC of 0.826 to 0.852. An SVM or radiomics score is a novel concept in clinical applications. An individualized SVM (radiomics) score could be calculated based on selected features (43–45). Xu et al. (43) used SVM score to preoperative lymph node metastasis in intrahepatic cholangiocarcinoma, with AUC of 0.87. Liu et al. (44) reported excellent performance in SVM score for prediction of treatment response in locally advanced rectal cancer, with AUC of 0.98. Park et al. (45) reported radiomics score improved the performance in MR prognostic model for glioblastoma. Zheng et al. (46) reported radiomics score is an independent prognostic factor for the postoperative outcome in solitary hepatocellular carcinoma. These studies suggest that radiomics features might be a useful tool in predicting recurrences in NFPAs, but no reports regarding this concept have been published as of yet.

To the best of our knowledge, preoperative radiomics approach for prediction of P/R in NFPAs is rarely reported. The SVM algorithm was utilized for feature selection and classification in this study. Three selected features were T1 surface-to-volume ratio, T1 GLCM-informational measure of correlation, and T2 NGTDM-coarseness. The surface-to-volume ratio is a shape index related to tumor infiltration. T1 GLCM-information measure of correlation is a texture feature related to the joint probability occurrence of the pixel pairs entropy. If the distribution of the intensities is more homogeneous, the value of this feature can be higher. T2 NGTDM-coarseness is an inverse measure of the level of the spatial rate of change in intensity. A higher value indicates a lower spatial change rate and a locally more uniform texture (47). In this study, three ROI methods were implemented, including the original tumor mask and two masks with differential erosion of the boundary pixels. The goal was to evaluate whether the potential inclusion of normal pituitary glandular tissue and other surrounding, non-tumorous structures would affect the prediction. The obtained results, however, turned out to be similar. One possible reason was that the eroded pixels were minimal compared to the whole tumor mask, thus accounting for the minimal overall effects on produced results.

In recent years, study of computer-extracted imaging radiomic features has become an active research field. However, the robustness and reproducibility of the selected quantitative imaging features need to be extensively studied before their clinical applications. Factors affecting the robustness of radiomic approach are modality dependent. So far only few studies have investigated the robustness of radiomic features in MRI (48–51). How different imaging sequences and imaging parameters will affect the reproducibility of radiomic features is still not clearly known. A recent phantom study noted that remarkable differences exist among different MRI sequences in the number of robust and reproducible features (52). Nevertheless, more than 30% (15 of 45) features still showed excellent robustness across all sequences and demonstrate excellent reproducibility. It was supposed that these 15 features can reliably be applied for the design of radiomics signatures within clinical studies. Among these features, the shape-related feature was noted to be robust. Another study of repeatability and reproducibility of MRI-based radiomic features also showed that shape features emerged as the most stable features among all the selected features (53). It was suggested that radiomics extracted from T1W and T2W imaging should be used with caution, and only robust and reproducible features should be selected for building a radiomics signature (52). However, it was also true that through fully automatic image segmentation as our study did, the effect of operators’ dependent bias of radiomic features can be reduced (52).

There were 41 true and 9 false predictions using the model developed with the original tumor mask. For most TP and FN cases, large tumor sizes with heterogeneous enhancement and surrounding bone invasion were observed. In contrast, small tumor sizes without bone invasion were found in most TN cases. Homogeneous contrast enhancement without apoplexy or cystic change was observed in most FP cases. Based on our results, macrocystic components or apoplexy may be an important factor leading to FN. Further studies involving a larger sample size is necessary to establish a better understanding regarding factors related to true and false predictions.

It is known that the extent of tumor resection is an important determining factor affecting recurrence rates in NFPAs (11). Although no statistical difference is demonstrated between GTR and P/R in our study, it may be due to the relatively small sample size. In our study, tumor recurrence was present in three patients despite having undergone GTR. In contrast, stable disease was observed in 16 patients after receiving STR only. Since most NFPAs are benign tumors, preoperative prediction of P/R in NFPAs offers clinically valuable information regarding treatment options. On the other hand, a significant correlation between the number of surgical resections and complication rates in NFPAs is reported (54). Anterior pituitary insufficiency and diabetes insipidus are the most commonly encountered postoperative complications in NFPAs with occurrence rates of 19.4 and 17.8% respectively (54). For patients with high possibilities of tumor recurrence, aggressive resection combined with postoperative adjuvant RT and close MR imaging follow-up should be considered. In contrast, for patients with lower possibilities of disease recurrence, the aim of surgery would be to relieve clinical symptoms by decreasing tumor mass effects. Optimal surgical planning for low risk patients could reduce potential complications of endocrine disorders while maintaining a good treatment outcome.

It is known that postoperative adjuvant RT offers excellent tumor control in 96% of patients with non-secreting adenomas (55). However, whether postoperative RT is beneficial for patients with low possibility of recurrence is controversial because RT may increase risks of complications such as visual deterioration, hypopituitarism, cerebrovascular accident, and dementia in NFPAs (55, 56). Because adjuvant RT may affect the independent predictive value of the preoperative MR radiomics analysis for P/R, patients who have received adjuvant RT before P/R were excluded from our study.

The study had several limitations. Selection bias may exist due to its retrospective nature. All MR images were acquired at a single site with a single protocol, and lack of external validation. Future testing with multi-institutional data and varying imaging protocols is necessary to determine whether the trained classifier is generalizable. Due to the relatively small sample size, only a few imaging features can be selected to build the classification model in order to avoid over-fitting. More advanced statistical analysis methods that can take all clinical and imaging factors into account need to be considered in the future. When more cases become available, other machine learning strategies, such as a fully automatic convolutional neural network able to perform end-to-end learning may be applied to improve the performance of prediction.

## Conclusions

In summary, our preliminary study of MR radiomics analyses based on CE T1WI and T2WI in preoperative MRI was able to achieve an accuracy of 82% and AUC of 0.78 in predicting recurrence in NFPAs. For SVM score based on selective features, an AUC of 0.87 was obtained in differentiation of P/R. The features extracted based on automatic segmentation and imaging registration were objective and quantitative. Because the robustness and reproducibility of MR radiomic features may be affected by imaging sequences and imaging parameters, more studies in this field are needed to know which reproducible radiomic features can be consistently used across imaging sequences and different institutions. The results in our study offer useful clinical information to aid in the preoperative as well as postoperative planning in the management of NFPAs, such as the extent of surgical resection, implementation of postoperative adjuvant RT, and the time interval of MR imaging follow-up. Nevertheless, this approach still needs to be validated with a larger-scale study and long-term follow-up.

## Data Availability Statement

The original contributions presented in the study are included in the article/**Supplementary Material**. Further inquiries can be directed to the corresponding author.

## Ethics Statement

The studies involving human participants were reviewed and approved by Chi Mei Medical Center Institutional Review Board (IRB no. 10902-009). Written informed consent for participation was not required for this study in accordance with the national legislation and the institutional requirements.

## Author Contributions

Conceived and designed the experiments: C-CK, J-HC. Performed the experiments: C-CK, YZ. Analyzed the data: C-CK, YZ, J-HC, K-TC, T-YC, S-WL. Contributed reagents/materials/analysis tools: T-YC, Y-KT. Wrote the paper: C-CK, YZ. Critically revised the article: J-HC, M-YS. All authors contributed to the article and approved the submitted version.

## Conflict of Interest

The authors declare that the research was conducted in the absence of any commercial or financial relationships that could be construed as a potential conflict of interest.
