# Supplementary material for: Radiomics Approach for Prediction of Recurrence in Non-Functioning Pituitary Macroadenomas
Source: Front Oncol. 2020 Dec 18;10:590083. doi: 10.3389/fonc.2020.590083 (PMC7775655; doi:10.3389/fonc.2020.590083)
Supplement: Supplementary file 1 [file DataSheet_1.docx]

**MRI Protocols**

The protocols of 1.5T (Siemens, MAGNETOM Avanto) MR imaging were as the following: axial T1-weighted imaging (T1WI) (spin-echo) (TR/TE, 2000/7 ms; FOV, 16-22 cm; slice thickness/spacing, 5 mm/6.5 mm; matrix, 320 x 224), sagittal T1WI (TR/TE, 2000/7 ms; FOV, 23 cm; slice thickness/spacing, 3 mm/3 mm; matrix, 320 x 224), axial T2-weighted imaging (T2WI) (fast spin-echo)(3670/108 ms; FOV, 16-22 cm; slice thickness/spacing, 5 mm/6.5 mm; matrix, 384 x 261), coronal T2WI (fast spin-echo)(3910/108 ms; FOV, 22 cm; slice thickness/spacing, 3 mm/3 mm; matrix, 384 x 270; voxel size, 0.57 x 0.81 x 3 mm^3^), axial fluid attenuated inversion recovery (FLAIR) (10000/106 ms; FOV, 16-22 cm; slice thickness/spacing, 5 mm/6.5 mm; matrix, 320x 196), and axial T2*-weighted gradient-recalled echo (GRE) (830/26 ms; FOV, 16-22 cm; slice thickness/spacing, 5 mm/6.5 mm; matrix, 256 x 168). Dynamic contrast-enhanced (CE) coronal T1WI images with small field of view through the pituitary gland (400/10 ms; FOV, 12 cm; slice thickness/spacing, 3 mm/3 mm; matrix, 256 x 205), as well as sagittal and coronal CE T1WI with fat saturation (2110/7 ms; FOV, 22 cm; slice thickness/spacing, 3 mm/3 mm; matrix, 256 x 207; voxel size, 0.86 x 1.06 x 3 mm^3^), were performed after intravenous administration of 0.1 mmol/kg of body weight of gadobutrol (Gadovist; Schering, Berlin, Germany) or gadoterate meglumine (Dotarem; Guerbet, Villepinte, France).

The protocols of 1.5T (GE Healthcare, Signa HDxt) MR imaging were as following: axial T1WI (spin-echo) (TR/TE, 2019/9 ms; FOV, 16-22 cm; slice thickness/spacing, 5 mm/6.5 mm; matrix, 320 x 192), sagittal T1WI (TR/TE, 1739/9 ms; FOV, 22 cm; slice thickness/spacing, 3 mm/4 mm; matrix, 320 x 192), axial T2WI (fast spin-echo)(3733/108 ms; FOV, 16-22 cm; slice thickness/spacing, 5 mm/6.5 mm; matrix, 320 x 224), coronal T2WI (fast spin-echo)(3567/100 ms; FOV, 22 cm; slice thickness/spacing, 3 mm/3 mm; matrix, 320 x 192; voxel size, 0.69 x 1.15 x 3 mm^3^), axial FLAIR (9002/116 ms; FOV, 16-22 cm; slice thickness/spacing, 5 mm/6.5 mm; matrix, 288x 192), and axial T2*-weighted GRE (517/20 ms; FOV, 16-22 cm; slice thickness/spacing, 5 mm/6.5 mm; matrix, 256 x 160). Dynamic contrast-enhanced (CE) coronal T1WI images with small field of view through the pituitary gland (400/8 ms; FOV, 18 cm; slice thickness/spacing, 3 mm/4 mm; matrix, 256 x 192), as well as sagittal and coronal CE T1WI with fat saturation (1966/9 ms; FOV, 22 cm; slice thickness/spacing, 3 mm/3 mm; matrix, 320 x 192; voxel size, 0.69 x 1.15 x 3 mm^3^), were performed after intravenous administration of 0.1 mmol/kg of body weight of Gadovist or Dotarem.

The protocols of 3T (GE Healthcare, Discovery MR750) MR imaging were as following: axial T1WI (spin-echo) (TR/TE, 3034/24 ms; FOV, 16-22 cm; slice thickness/spacing, 5 mm/6.5 mm; matrix, 352 x 224), sagittal T1WI (TR/TE, 2316/22 ms; FOV, 22 cm; slice thickness/spacing, 3 mm/3 mm; matrix, 320 x 224), axial T2WI (fast spin-echo)(5152/102 ms; FOV, 16-22 cm; slice thickness/spacing, 5 mm/6.5 mm; matrix, 384 x 320), coronal T2WI (fast spin-echo)(4353/106 ms; FOV, 22 cm; slice thickness/spacing, 3 mm/3 mm; matrix, 384 x 256; voxel size, 0.57 x 0.86 x 3 mm^3^), axial FLAIR (10000/95 ms; FOV, 16-22 cm; slice thickness/spacing, 5 mm/6.5 mm; matrix, 320x 192), and T2*-weighted GRE (567/20 ms; FOV, 16-22 cm; slice thickness/spacing, 5 mm/6.5 mm; matrix, 256 x 192). Dynamic contrast-enhanced (CE) coronal T1WI images with small field of view through the pituitary gland (571/14 ms; FOV, 12 cm; slice thickness/spacing, 3 mm/3 mm; matrix, 224 x 160), as well as sagittal and coronal CE T1WI with fat saturation (2494/18 ms; FOV, 22 cm; slice thickness/spacing, 3 mm/3 mm; matrix, 320 x 224; voxel size, 0.69 x 0.98 x 3 mm^3^), were performed after intravenous administration of 0.1 mmol/kg of body weight of Gadovist or Dotarem.
